# Sleep Disorders in Childhood Neurological Diseases

**DOI:** 10.3390/children4100084

**Published:** 2017-09-22

**Authors:** Abdullah Tolaymat, Zhao Liu

**Affiliations:** Department of Pediatrics, Division of Pediatric Neurology, Children’s Hospital of Illinois, University of Illinois College of Medicine at Peoria, 520 N.E. Glen Oak Ave, Peoria, IL 61637-0001, USA; liuzh@uicomp.uic.edu

**Keywords:** sleep disorders, sleep disordered breathing, neurological diseases, neuro-developmental conditions

## Abstract

Sleep problems are frequently addressed as a primary or secondary concern during the visit to the pediatric neurology clinic. Sleep disorders can mimic other neurologic diseases (e.g., epilepsy and movement disorders), and this adds challenges to the diagnostic process. Sleep disorders can significantly affect the quality of life and functionality of children in general and those with comorbid neurological diseases in particular. Understanding the pathophysiology of sleep disorders, recognizing the implications of sleep disorder in children with neurologic diseases and behavioral difficulties, and early intervention continue to evolve resulting in better neurocognitive outcomes.

## 1. Introduction

Sleep disorders are commonly addressed in pediatric neurology practices and often present as a chief complaint or as a secondary concern concomitant with a wide range of neurological diseases and neurogenetic syndromes. About 25% of children may have at least one sleep problem by adolescence and reaches up to 75% in those with epilepsy, attention deficit hyperactivity disorder (ADHD), Autism Spectrum Disorder, or headaches [1,2,3,4,5].

Sleep disorders alter the normal sleep architecture leading to a disrupted sleep and lower sleep quality, which in turn may increase headaches frequency, complicate epilepsy management, and cause emotional lability, hyperactive behavior, or impaired cognition. Sleep disorders can be primary, such as narcolepsy and insomnia, or secondary to neurologic diseases, whether central in origin as in central sleep apneas or peripheral obstructive from neuromuscular diseases.

In this review, we will provide a focused overview of sleep disorders in a number of neurologic diseases and neurobehavioral conditions and their implications on the cognitive functioning in these children.

## 2. Sleep-Wake Cycles Regulation

**Figure 1:**
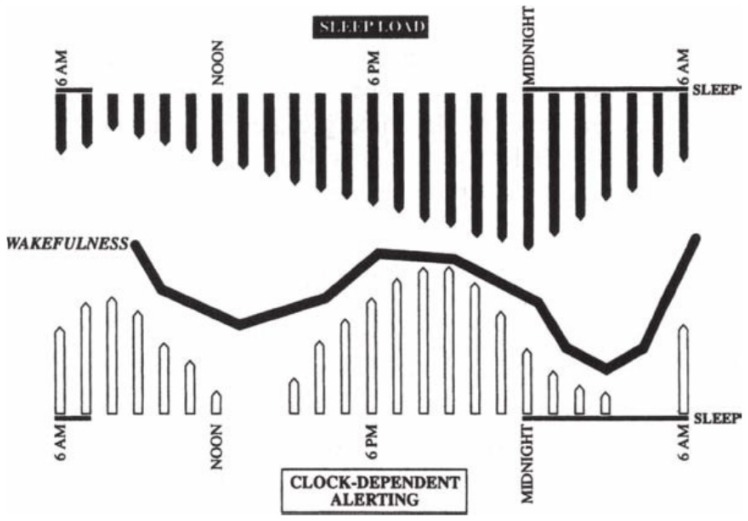
Normal distribution of sleep stages in healthy children, adults, and the elderly [6].

Sleep and wakefulness are regulated by two simultaneous processes: a homeostasis process and an endogenous process, illustrated in Figure 1 [6].

Homeostatic process “process S” primarily regulates the length and depth of sleep resulting from the accumulation sleep-promoting agents such as adenosine and cytokines during prolonged periods of wakefulness, and this tends to build up more quickly in infants and young children. Endogenous circadian rhythm or “process C” helps with sleep organization, timing, and the duration of daily sleep and wake cycles “master circadian clock” primarily by suprachiasmatic nucleus (SCN) in the ventral hypothalamus, which also plays a physiologic rule in other body systems (e.g., hormone levels, renal, cardiovascular and pulmonary functions). The neurodevelopmental maturation and the environmental factors (light–dark cycles) help the circadian rhythm to rapidly develop early in infancy.

Sleep propensity or alertness during a 24 h period is partially determined by the duration and quality of the previous sleep and wakefulness. Circadian rhythms are synchronized to the 24h day cycle by environmental cues like the light–dark cycle, which is the most important cue as light signals are transmitted to the SCN via the circadian photoreceptor system within the retina which then stimulates (with darkness) or inhibits (with light) melatonin production by the pineal gland [7,8,9,10].

This brief review of sleep-wake cycles’ regulation represents the significant impact that neurologic diseases may have on sleep initiation, maintenance, and organization.

## 3. Central Nervous System Malformations

Children with Arnold–Chiari malformation may experience central sleep apnea due to brainstem compression that affects the respiratory drive, but they may also have obstructive sleep apnea (OSA) due to denervation or activation of the nerves that innervate pharyngeal and laryngeal muscles that contributes to a narrower upper airway.

Meningomyelocele (specifically thoracic and thoracolumbar) and Chiari malformation type II can be associated with blunted ventilatory and arousal responses, central hypoventilation syndrome, and increased central and obstructive apneas [11].

Surgical decompression of the posterior fossa in Chiari malformation or tethered cord release in children with meningomyelocele may be necessary. Adenotonsillectomy can also improve the obstructive disease, but positive pressure mechanical ventilation with continuous positive airway pressure or bi-level positive airway pressure is often needed, especially in children whose respiratory function is further compromised by kyphoscoliosis or obesity.

Joubert’s syndrome is associated with characteristic respiratory panting of episodic tachypnea occurring during wake or sleep immediately followed by apneic episodes lasting up to 1 minute, often without associated bradycardia or cyanosis [12].

## 4. Achondroplasia

Achondroplasiais the most common cause of dwarfism with short limbs. Sporadic mutation in is found in the vast majority of cases but it can also be inherited an autosomal dominant disorder. Achondroplasia is caused by a mutation in fibroblast growth factor receptor 3 (FGFR3), which normally has a negative regulatory effect on bone growth. Achondroplasia is characterized by short stature with short arms and legs, craniofacial deformities such as macrocephaly, frontal bossing, small foramen magnum, short cranial base, and severe midface hypoplasia [13].

Sleep-related breathing disorders are found in about 75% of achondroplasia patients ranging from OSA with significant oxygen desaturations, obstructive hypopneas, to continuous and loud snoring [14]. Twenty-five percent show rare central apnea events during REM. OSA may occur early in life due to the midface hypoplasia and macroglossia. Although achondroplasia is not primarily a neurologic condition, some patients have a degree of compression at the cervicomedullary junction that may result in central sleep apnea.

In another study, different groups of children with achondroplasia were identified based on the severity of the sleep disorders found:mild, presenting only with obstructive sleep apnea due to midfacial hypoplasia causing upper airway (UA) obstruction (and adenotonsillar hypertrophy); moderate, with obstructive sleep apnea (OSA) due to upper airway muscle weakness along with hydrocephalus (due to a small foramen magnum); and severe, with upper airway obstruction, cor pulmonale, severe respiratory failure, and death [15].

## 5. Autism Spectrum and Neurodevelopmental Disorders

The autism spectrum disorders (ASD) include a wide continuum of disorders remarkable for the associated cognitive, neurobehavioral deficits, socialization, and communication deficits.

There is high association between autism spectrum disorders (ASD) and sleep problems, which add more difficulties to children with ASD. Sleep concerns and difficulties usually start to arise beginning in infancy even before the autismspectrum disorder diagnosis is officially given. A study assessed sleep problems at two age ranges (7–9 and 11–13 years) revealed that chronic insomnia wasmore than ten times higher in ASD children than that observed in controls [16].

Another study showed that more than half of the families ofchildren with ASD (57.6%) reported sleep problems, including insomnia, fragmented sleep, night terrors, and early morning waking [17]. Their stereotypic and repetitive behaviors and the difficulties to adapt to any change in their daily routine play a role in their sleep problems, which in turn directly or indirectly contributes to their impaired cognition and neurodevelopmental deficits. Wiggs and Stores found that the sleep disorders were most commonly behavioral as in the behavioral insomnia of childhood—sleep-onset type and insomnia due to Pervasive Developmental Disorders and it was noted that children with ASD may wake up at night without alerting their parents, which they called “contented sleeplessness,” contrary to other children who may wake up and go to their parents’ bedroom [18].

A single-night PSG study was done on a group of children with ASD and found a lower rapid eye movement (REM) sleep percentage compared with both children with normal development and children with other developmental disorders [19]. REM sleep is greatest in the developing brain and may represent a protected time for neuroplasticity and is involved in memory consolidation, normal cognitive function, and the processing of emotional memory [19,20,21].

There could be many pathophysiologic mechanisms underlying the insomnia. To begin with, children with autism have difficulties with emotion self-regulation, comprehension, compliance with the parental instructions and with expressing their apprehensions [22,23]. Children with ASD have fewer recollections of dreams and distinctive slower alpha EEG patterns and asymmetries [24,25].

Abnormal platelet serotonin level is one of the few consistent biochemical findings in children with autism [19,26]. Serotonin is a precursor to melatonin and genetic abnormalities have been reported in the melatonin pathway including acetylserotonin-*O*-methyltransferase (ASMT), an enzyme responsible for the synthesis of melatonin from serotonin found in a small number of children with ASD [27].

About 30% of children with autism have epilepsy [28], and seizures are often activated in sleep and that usually warrants an appropriate work up for uncharacterized nocturnal spells by obtaining EEG and polysomnography. A detailed clinical history supported by a video clip recorded by family members is sometimes sufficient to differentiate between sleep-related behavior and nocturnal seizure disorder.

Rhythmic movement disorder (RMD), such as head banging, can be seen at night or unrelated to sleep during the day. Low iron stores are seen in children with ASD due to their eating habits and incompliance with balanced diets, and they become susceptible to restless leg syndrome. Iron supplementation was shown to improve restless sleep in children with ASD [29].

Management primarily consists of optimizing sleep environment and appropriate sleep hygiene. Few studies focused on the effects of behavioral interventions and melatonin. The Melatonin in Children with Neurodevelopmental Disorders and Impaired Sleep (MENDS) study [30] was a randomized clinical trial (RCT) that studied the effect of an escalated dose of melatonin on total sleep time over 3-month period on randomized 146 children, 63 of them had autism and learning difficulties, and the rest had other non-specific causes of learning difficulty. A standardized parental sleep behavioral intervention [31] was used for a month prior to melatonin use during which a routine bedtime was established. More than 50% of the enrolled subjects improved to the degree that some subjects were not qualified to continue for the pharmacological RCT.

Cortesi et al. [32] study showed that behavioral intervention in addition to melatonin treatment result in a better treatment response. The pharmacological placebo-controlled intervention showed a small increase of 23 min in total sleep time (TST) and a 38 min reduction in sleep latency with the administration of 0.5–12 mg immediate-release melatonin in a stepwise fashion but showed no remarkable effects on night waking. Clonidine, trazodone, and atypical antipsychotic medications can improve sleep for children with ASD as reported in few case series and open-label studies [33,34].

Rett’s syndrome is a neurodevelopmental disease that primarily affects females and is the second most common cause of mental retardation in females after trisomy 21 syndrome, with an incidence of 1/10,000–22,000 females [35,36]. This syndrome is remarkable for an initial normal development followed by developmental regression more significant for speech, microcephaly, emergence of stereotypic wringing hand movements, autistic features, and often a seizure disorder. The so far known genes that lead to such phenotypes are *MECP2* (Xq28), the *CDKL5* (Xp22) [37,38,39], the *Netrin G1* (chromosome 1) [39], and the *FOXG1*gene (14q12) [41]. The MECP2 protein may have a role in the maintenance of neurons in the developing brain.

Children with Rett’s syndrome have a wide variety of sleep-related abnormalities including irregular breathing, bruxism, sleep disturbances, and night laughter. A questionnaire involved 202 patients with Rett’s syndrome in 2004 revealed a high prevalence of sleep problems in about 80–94% of these patients and reported night laughing, teeth grinding, night screaming and nocturnal seizures as the most frequent encountered sleep disturbances in the studied group [42].

The breathing irregularities can consist of hyperventilation or breath-holding and may occur during wakefulness [43,44]. Hyperventilation can be interrupted by breathing pauses and apneas of about 30–40seconds. The opiate antagonist naltrexone was considered as a treatment for breathing irregularity on a trial basis only, but no significant benefits were proved [45].

## 6. Neuromuscular Diseases

Sleep-disordered breathing (SDB) is frequently encountered in neuromuscular diseases including Duchenne muscular dystrophy, Charcot-Marie-Tooth disease, myotonic dystrophy, congenital myopathies, and many others. Such diseases may involve the respiratory muscles (diaphragm, intercostal muscles, and accessory respiratory muscles). These patients may also have reduced vital capacity secondary to scoliosis, which may further compromise the respiratory function and exacerbate or complicate SDB.

The risk of desaturation or hypoxemia is greatest during REM sleep [46,47]. Patients with neuromuscular disorders are more susceptible to the disordered breathing during REM sleep due to the physiologic atonia which affects the upper airway and chest wall accessory breathing muscles and spares the function of the diaphragm. Thus, if these patients have upper airway obstruction, central apneas occur even though they seem obstructive in origin (pseudocentral apnea) [48,49,50].

Obstructive or central apneas together with hypoventilation are the most common described sleep abnormalities in children with neuromuscular disorders. The involvement of bulbar, diaphragm, and intercostal muscles may result in an airway obstruction, which is remarkably seen in the transition from sleep to wake state.

If the disease affects the diaphragm, hypoventilation and central sleep apnea may occur in the recording due to the lack of respiratory effort. Early in the course of a neuromuscular disease, sleep is not disordered in NREM; however, in an advanced stage, hypoventilation may occur during NREM sleep which is an alarming sign for possible hypoventilation during wake state as well [46,47]. Patient’s age, disease type, progression, and the involvement of the upper airway and respiratory muscles usually determine the severity of sleep disorders.

Duchenne’s muscular dystrophy (DMD) and related sleep and breathing disorders are widely studied where central apnea, OSA, hypoventilation and oxygen desaturation are frequently observed [51]. However, significant SDB can occur even in mild respiratory muscle weakness. The hypoxemia and chronic respiratory failure in these patients is usually preceded by periods of increased end-tidal CO_2_ during sleep with normal daytime blood gases. Pulmonary function tests help to assess maximal inspiratory and expiratory pressures and the severity of muscle weakness.

## 7. Sleep and Epilepsy

The interictal epileptiform discharges are often activated during sleep, and the propensity to have clinical seizures is also increased.

Sleep disruption with epilepsy can be multifactorial secondary to, for example, nocturnal seizures that may disrupt sleep organization and or antiepileptic medications, their effects and adverse reactions. Other neurodevelopment comorbidities increase the likelihood of sleep disorders in children with epilepsy.

Frontal and temporal lobe seizures are more likely to occur and to generalize secondarily during sleep [52].

An electroclinical syndrome called electrical status epilepticus during slow-wave sleep (ESES) is characterized by continuous spike-and-wave discharges during most of the nocturnal NREM sleep, combined with cognitive and behavioral regression like in Landau Kleffner syndrome [53]. Liukkonen et al. longitudinally followed 32 children with ESES treated with valproic acid alone or valproic acid with ethosuximide. Ten of 32 children regained their baseline pre-ESES cognitive levels [54]. In general, this condition is intractable to epilepsy pharmacotherapy and may have unfavorable cognitive outcomes.

Several pediatric epilepsy syndromes are also known for their nocturnal activation such as benign rolandic epilepsy and benign occipital epilepsy of childhood.

Sleep disorders can adversely affect seizure control. Sleep deprivation leads to activation of seizures even in healthy individuals [55].

Epileptic patients with obstructive sleep apnea OSA have shown to have better seizure control after the correction of OSA with positive airway pressure breathing, adenotonsillectomy, or tracheostomy [56].

On the other hand, optimal seizure control helps sleep quality byminimizing sleep disruption caused by nocturnal arousals or seizures.

## 8. Cerebral Palsy

Cerebral palsy is a group of disorders of movement, tone, and posture resulting from an insult to the developing fetal or infant brain. The motor disorders of cerebral palsy are often accompanied by intellectual disabilities, sleep disturbances, epilepsy, or any combination of these three.

Structural brain lesions in children with cerebral palsy may directly or indirectly lower sleep quality due to sleep disruption, difficulties in sleep initiation, or sleep apnea. Children with cerebral palsy are at higher risk for central apnea secondary to the brain injuries and the disrupted and dysfunctional sleep–wake cycle pathways. They may also have obstructive sleep apnea not only because of hypertrophic tonsils and adenoids; the disproportionate midface anatomy, mandibular alterations and abnormality of the upper airway tone (hypotonia, hypertonia, or dystonia), medication effect that may alter the tone of the upper air way are also factors that contribute to obstruction and thus cause subjects to exhibit daytime irritability or excessive sleepiness due to fragmented sleep, frequent awakenings, and oxygen desaturation [57,58,59].

They may also have impaired central arousal mechanisms to compensate for apnea by body position changes, and this increases the significance of their obstructive sleep apnea (OSA).

Looking at the prevalence of respiratory disturbances during sleep from a questionnaire-based survey on 233 children with cerebral palsy; snoring in 63% and sleep apnea in 19.7% were reported. Forty-eight of these children underwent a screening sleep study and found that 27% of these 48 children have an apnea–hypopnea index (AHI) >5 and 58% had SaO_2_ less than 85% [60].

A study that investigated sleep organization in 23 children with cerebral palsy showed that 12 out of 23 patients had abnormal sleep EEG architecture, such as the absence of EEG characteristics of NREM sleep and REM sleep, a low incidence, or an extreme presence of sleep spindles and high percentage of early awakenings after sleep onset [61].

Children with cerebral palsy may also have circadian rhythm disorders (irregular sleep–wake rhythms), especially when experiencing associated blindness that alters light perception, which affects melanopsin activation in the retinal ganglionic cells and melatonin secretion from the pineal gland that regulates circadian rhythms [62]. Melatonin and bright light (if there is intact light perception) may be helpful to reset and normalize sleep schedules.

Pain and discomfort that children with cerebral palsy may experience also affect the sleep to a large extent. This was shown to have the strongest negative impact on sleep in a study of a population of children with physical disabilities [63,64]. A study performed on 26 children with quadriplegic cerebral palsy who received Botulinum toxin type A injections for lower limb spasticity, and improved sleep with less sleep disruption and a decreased need for turning because of discomfort were reported [65].

A study conducted on 35 children with cerebral palsy and severe spasticity showed a decrease in the frequency of nighttime awakening and severity of pain in 6 months following the implantation of an intrathecal baclofen pump [66].

## 9. Headaches

Migraine is a common form of primary headaches that may occur early in childhood. The prevalence of migraine in children is estimated about 10%, with higher rates reported among teenagers [67,68,69,70]. Tension-type and nonspecific recurrent headaches have been reported in 2–3% of children [71].

Sleep disruption and headaches are strongly related, and a disordered sleep may result in headaches and headaches may cause sleep problems such as insomnia and parasomnias. Both conditions highly increase the risk of each other. The pathophysiology of this relationship between migraines and sleep disruption has been thought to involve hypothalamic-mediated decreases in serotonin and alternations in melatonin secretion. Sleep disorders are noted to be frequent comorbid disorders in children with migraines, followed by anxiety disorders and depression [72].

Sleepwalking has also been reported in different groups of migraine patients, in about 21.9–30% of cases [73,74,75]. A study in school age children showed that there is higher prevalence of sleep disorders such as insomnia, frequent night awakenings, parasomnias (mainly sleep talking, sleep walking and bruxism), and enuresis in children with migraines or tension headaches [74,76,77]. Children with OSA may complain of headaches upon waking in the morning, which could be related to hypoxia or elevated CO_2_ levels [78]. Headache and sleep diaries, along with polysomnography, are very useful tools to precisely find a link and correlation between headaches and sleep problems, especially in the presence of suspected sleep disorder that needs to be further studied and investigated like insomnia, parasomnias, or OSA. Additionally, an actigraph, which is a wrist-worn device of watch size that measures movement over time as an indirect indicator of sleep and wakefulness, is often used in such cases. It can record sleep parameters such as total sleep, sleep onset latency, total time in bed (from lights out to getting out of bed), and sleep efficiency (ratio of total sleep duration to total time spent in bed) [79]. Actigraphy is useful for monitoring circadian rhythms and sleep disturbances in children as indicated by the Standards of Practice Committee of the American Academy of Sleep Medicine (AASM) [80] and is successfully used to monitor sleep and observe sleep patterns of children with chronic pain conditions, including migraines.

l-5-Hydroxytryptophan in migraine cases has shown improvement in headache and sleep disorders in migraine patients, specifically in frequent night awakenings and parasomnias [81]. A small study included 35 children with migraines whose sleep–wake rhythm was behaviorally regulated by following sleep hygiene guidelines, showed a remarkable reduction in headache duration and frequency [82].

## 10. Conclusion

In conclusion, sleep disorders are commonly encountered in children with comorbid neurological diseases but may not be addressed by the referring physician or caregivers leading to unidentified sleep problems. Assessing sleep history is always recommended to recognize major sleep problems that may warrant further investigation and management. Treating sleep disorders may remarkably help improving the outcome of the treated neurological diseases and the cognitive function of these patients.
